# Knowledge, attitudes, and practices of oral and maxillofacial surgeons towards the use of artificial intelligence in clinical practice and training: a cross-sectional study

**DOI:** 10.3389/froh.2025.1630995

**Published:** 2025-09-23

**Authors:** Bernadette Quah, Chee Weng Yong, Matthias Wei Jin Chen, Clement Wei Ming Lai, Intekhab Islam

**Affiliations:** 1 Faculty of Dentistry, National University of Singapore, Singapore, Singapore; 2 Discipline of Oral and Maxillofacial Surgery, National University Centre for Oral Health, Singapore, Singapore

**Keywords:** artificial intelligence, surgery, oral, health knowledge, attitudes, practice, health care surveys, health education

## Abstract

**Aim:**

This study aimed to evaluate the knowledge, attitudes, and practices of oral and maxillofacial surgery (OMS) clinicians and trainees relating to the use of artificial intelligence (AI) within OMS practice and training.

**Methods:**

A cross-sectional survey study was conducted with OMS specialists and trainees in Singapore regarding their views on AI in OMS. The survey comprised 25 questions over five sections, and was distributed via an online survey platform.

**Results:**

48 participants completed the survey, including 37 specialists and 11 trainees. 60.4% did not report a good understanding of AI, 52.1% were not aware of the uses of AI in OMS, and 81.3% had not had any form of AI-related training. Most felt that AI could be beneficial for diagnosis and treatment planning (72.9%) and enhancing patient outcomes (75.0%), and should be incorporated into OMS training (68.8%). While there were no differences between genders, younger participants tended towards more positive attitudes (*p* < 0.05). Participants cited concerns about inaccurate diagnoses or plans (77.1%), overdependence (70.8%), privacy/security concerns (41.7%), and increased healthcare costs (41.7%). Although most participants reported using AI in daily life (68.8%) and noted that AI made the completion of tasks easier (62.5%), most have not incorporated AI into their clinical practice (62.5%), and felt that inadequately trained or equipped to do so (79.2% and 58.3% respectively).

**Conclusion:**

OMS specialists and trainees in Singapore generally have optimistic views toward AI, with younger respondents tending towards more positive attitudes. The levels of knowledge and practice leave room for improvement.

## Introduction

Artificial intelligence (AI) technologies have been emerging in the healthcare scene in recent years. In academia, the volume of AI-related publications in medicine has consistently increased, with an annual growth rate of 28.4% (1). The field of oral and maxillofacial surgery (OMS) has seen similar trends, with AI-driven models being developed to aid both diagnosis and surgical treatment planning (2). Tech giants like Google have followed suit to increase the scale of such developments, creating models like MedLM to answer medical questions and AI-driven tools to detect pathology (3). In healthcare education, a multitude of potential applications to improve education have also been proposed, including their use as teaching aids, self-directed learning platforms, and as a part of automated assessment (4).

While industries are pushing for the integration of AI into healthcare and education, the willingness for uptake of these new technologies on the ground by clinicians, educators, and students in their day-to-day work is less known. Knowledge, attitudes and practices (KAP) studies have gained widespread acceptance in the health sciences to understand baseline perspectives on specific topics and before program refinement and optimisation (5).

Relating to AI, several KAP studies of researchers, educators, students and healthcare professionals have been published in the past three years (6–8) In dentistry, while KAP studies including dental students and dentists have found variable levels of knowledge and generally favourable attitudes towards incorporating AI into the dental curriculum and dental practice (9). However, no studies specific to clinicians in OMS and few to no studies in the East or Southeast Asian populations have been performed. Beyond the use of AI in improving dental care, OMS is unique in its incorporation of medicine and surgery to broaden the scope of dentistry to include the hard and soft tissues of the face. It is thus unknown if the knowledge and attitudes of an OMS practitioner will be similar to those of another dentist.

Therefore, the objective of this study is to evaluate the knowledge, attitudes and current practices of OMS specialists and trainees regarding the use of AI in clinical practice and training.

## Materials & methods

A cross-sectional survey study was conducted among OMS clinicians in the public and private sectors in Singapore from 7 October 2024 to 15 November 2024. The survey was created in accordance with the Checklist for Reporting Results of Internet E-Surveys (CHERRIES) (10). Participants enrolled in the study included OMS specialists and trainees; OMS specialists were defined as clinicians registered as specialists in OMS by the Singapore Dental Council, while OMS trainees include residents who are currently enrolled in the National University of Singapore (NUS) Master of Dental Surgery (OMS) program (the only OMS residency program in Singapore), or clinicians who have completed an OMS residency program and who currently practice in Singapore without the specialist accreditation. Exemption from the National University of Singapore Institutional Review Board (IRB) was sought prior to data collection (NUS-IRB-2024-892).

### Survey development, testing and validation

An online survey platform (Qualtrics XM, USA) was used for survey hosting and data collection. The survey questions were formulated after discussion within the research team (Table 1). The survey was divided into five sections: Section 1 compiled the participants’ demographic information (age, gender, years of clinical practice), Sections 2, 3 and 4 assessed the knowledge, attitudes and practices of the participants respectively, and Section 5 was left open-ended for additional comments. A combination of multiple-choice questions, multi-selection questions, questions utilising the Likert scale and open-ended questions were incorporated.

**Table 1:** Survey sections and questions.

Section 1: Demographics	Answer Options				
Q1: What is your age?	______________				
Q2: What is your gender?	Male	Female			
Q3: Are you an accredited specialist in OMS?	Yes	No			
Q4: How many years have you practiced OMS?	0–5	6–15	>15		
Section 2: Knowledge					
Q1: I have a good level of understanding of artificial intelligence (AI), e.g., machine learning, large language models	Strongly Agree	Somewhat Agree	Somewhat Disagree	Strongly Disagree	
Q2: I am aware of the uses of AI in OMS practice and education	Strongly Agree	Somewhat Agree	Somewhat Disagree	Strongly Disagree	
Q3: I have attended courses, lectures or any other form of training in AI	Strongly Agree	Somewhat Agree	Somewhat Disagree	Strongly Disagree	
Q4: Please list any AI technologies you are familiar with (within or outside OMS)	__________________________________________				
Section 3: Attitudes					
Q1: AI is or can be beneficial in enhancing patient outcomes in OMS	Strongly Agree	Somewhat Agree	Unsure	Somewhat Disagree	Strongly Disagree
Q2: AI should be integrated in clinical practice for diagnosis and treatment planning	Strongly Agree	Somewhat Agree	Unsure	Somewhat Disagree	Strongly Disagree
Q3: AI should be a part of OMS training	Strongly Agree	Somewhat Agree	Unsure	Somewhat Disagree	Strongly Disagree
Q4: AI may replace OMS surgeons in the future	Strongly Agree	Somewhat Agree	Unsure	Somewhat Disagree	Strongly Disagree
Q5: Overuse of AI may cause surgeons to lose certain clinical skills	Strongly Agree	Somewhat Agree	Unsure	Somewhat Disagree	Strongly Disagree
Q6: What do you think are some advantages of AI in OMS practice and education? (you may select more than one option)	○ Increased clinical efficiency ○ Reduced workload for clinicians, educators or trainees ○ Increased accessibility and personalisation for patients/students ○ I do not think there are any advantages ○ Others: ______________				
Q7: What are your concerns regarding using AI in clinical practice? (you may select more than one option)	○ Privacy and security concerns ○ Inaccurate diagnosis or treatment ○ Clinician overreliance and eventually becoming obsolete ○ Increased healthcare costs ○ ○ Others: ______________				
Section 4: Practices					
Q1: I have used AI technologies in any field/other times in life outside of work	Strongly Agree	Somewhat Agree	Unsure	Somewhat Disagree	Strongly Disagree
Q2: I have used AI technologies in my practice of OMS	Strongly Agree	Somewhat Agree	Unsure	Somewhat Disagree	Strongly Disagree
Q3: I have used or considered using and for the following purposes: (you may select more than one option)	○ Diagnosis (radiographic, histopathologic, clinical) ○ Patient or student education ○ Self-directed learning ○ Treatment planning ○ Intraoperative as an adjunct ○ I have not used or considered using AI in my practice ○ Others: ______________				
Q4: I have used or considered using AI in the diagnosis or treatment planning for the following OMS subspecialties: (you may select more than one option)	○ Dentofacial deformities ○ Dentoalveolar surgery ○ Surgical pathology (including surgical oncology) ○ Maxillofacial trauma ○ Implant and preprosthetic surgery ○ TMJ surgery ○ I have not used or considered using AI in my practice				
Q5: AI makes completion of my work easier	Strongly Agree	Somewhat Agree	Unsure	Somewhat Disagree	Strongly Disagree
Q6: I feel adequately trained to use AI tools	Strongly Agree	Somewhat Agree	Unsure	Somewhat Disagree	Strongly Disagree
Q7: My clinic or institution is equipped to incorporate AI into clinical practice	Strongly Agree	Somewhat Agree	Unsure	Somewhat Disagree	Strongly Disagree
Q8: What resources do you think are necessary for better integration of AI in your practice?	__________________________________________				
Section 5: Other Comments					
Q1: Do you have any other comments?	__________________________________________				

Sample size calculation was performed prior to survey administration, and a target sample size of 52 was calculated for a 95% confidence level and 8% margin of error.

The sample was planned to be obtained using convenience sampling. Prior to dissemination, a qualitative assessment of the survey was performed to ensure validity. Content validity was verified via a review of the survey questions by a panel of three experts in the field of OMS and AI. These experts assessed the questions and gave feedback on the relevance and comprehensiveness of the survey. Subsequently, face validity was verified via pilot testing conducted with 6 participants (i.e., 10% of the target sample size). This was done as part of survey refinement to ensure question clarity, comprehensibility and relevance. The internal consistency of Sections 2, 3 and 4 (Knowledge, Attitudes and Practices respectively) was assessed using Cronbach's alpha, with a value above 0.7 considered as an acceptable level of internal consistency.

### Survey administration

The survey was distributed to potential participants via email through local professional organisations (the Association of Oral and Maxillofacial Surgeons Singapore, AOMSS) and academic institutions (NUS). The survey was kept open, with no password required to enter the survey. Participants were informed of the aims of the study, the study team members and the approximate duration required to complete the survey, and consent was obtained upon commencement of the survey. Participation was voluntary, and participants were given the option to withdraw at any point. All responses were confidential, and participants were not asked to provide identifiers. No incentives were provided to participants who completed the survey.

Data collected via the survey platform was exported on an Excel sheet. Apart from the survey responses, data on the number of unique users was tallied using IP checks and cookies to sieve out duplicate responses. Timestamps were also recorded to identify surveys that were completed in under 20 s and surveys which were not completed, which indicated an inaccurately filled-out survey that should be excluded.

### Data analysis

Descriptive statistics (frequencies and percentages) were computed for each survey question. In addition to this, statistical analyses were performed to identify any associations between demographic factors (i.e., gender, age, years of practice) and survey responses. For questions following the Likert scale, positive responses (“Strongly Agree” and “Somewhat Agree”) were combined to form one variable “Agree”, while negative responses (“Strongly Disagree” and “Somewhat Disagree”) were combined to form one variable “Disagree”. Questions that followed a five-point Likert scale had an additional response option “Unsure”, which was included as a third variable. The Fisher's exact test was then used to assess for associations between the responses and demographic factors. Similarly, for questions allowing multiple responses, each response option was treated as a separate binary variable (selected vs. not selected), and the Fisher's exact test was then performed to determine whether the proportion of each option differed between any demographic groups. All statistical analyses were conducted using the R Statistical Software, with the significance level set at *p* < 0.05.

Lastly, thematic analysis of the free-text responses to the open-ended questions in Section 4 and Section 5 was performed by members of the study team in phases, with data familiarisation followed by coding. The coded data were subsequently segregated into themes as deemed appropriate to explore the various themes of how to better integrate AI into practice.

## Results

55 responses were received by the stipulated deadline of 15 November 2024. Seven of these responses were incomplete and hence excluded, leaving a total of 48 valid responses included for analysis. Participants took a mean duration of 2.43 min to complete the survey. The mean age of the participants was 40.8 years, with 35 (72.9%) being male and 13 (27.1%) being female. There was a roughly equal distribution of participants between the three groups of years of practice: 15 (31.3%) participants had been practicing for 5 years or less (i.e., trainees), 17 (35.4%) had been practicing for 6-15 years (new/junior specialists), and 16 (33.3%) had been for more than 15 years (senior specialists).

### Knowledge

Overall, the majority of participants reported lacking knowledge in the field of AI in OMS (Table 2). 60.4% of respondents answered “Strongly Disagree” or “Somewhat Disagree” to having a good level of understanding of AI in general, 52.1% responded similarly to being aware of the uses of AI in OMS. Only 18.8% reported having attended some form of training related to AI. There was no significant difference in responses by gender (*p* = 0.741–1.000), age (*p* = 0.153–1.000) or number of years of practice (*p* = 0.222–1.000) (Table 3). The Cronbach's alpha of 0.76 for the knowledge section indicated an acceptable level of consistency (Table 4).

**Table 2:** Summary of responses to questions utilising the Likert scale.

Question	Strongly Agree	Somewhat Agree	Unsure	Somewhat Disagree	Strongly Disagree
Section 2: Knowledge					
Q1: I have a good level of understanding of AI	2 (4.2%)	17 (35.4%)	NA	19 (39.6%)	10 (20.8%)
Q2: I am aware of the uses of AI in OMS	4 (8.3%)	19 (39.6%)	NA	19 (39.6%)	6 (12.5%)
Q3: I have attended training in AI	1 (2.1%)	8 (16.7%)	NA	17 (35.4%)	17 (35.4%)
Section 3: Attitudes					
Q1: AI is or can be beneficial in enhancing patient outcomes	17 (35.4%)	19 (39.6%)	10 (20.8%)	1 (2.1%)	1 (2.1%)
Q2: AI should be integrated for diagnosis and treatment planning	15 (31.3%)	20 (41.6%)	9 (18.8%)	2 (4.2%)	2 (4.2%)
Q3: AI should be a part of OMS training	14 (29.2%)	19 (39.6%)	9 (18.8%)	4 (8.3%)	2 (4.2%)
Q4: AI may replace OMS surgeons in the future	0 (0%)	3 (6.3%)	7 (14.6%)	14 (29.2%)	24 (50.0%)
Q5: Overuse of AI may cause surgeons to lose certain clinical skills	6 (12.5%)	22 (45.8%)	5 (10.4%)	11 (22.9%)	4 (8.3%)
Section 4: Practices					
Q1: I have used AI technologies in any field	10 (20.8%)	23 (47.9%)	2 (4.2%)	8 (16.7%)	5 (10.4%)
Q2: I have used AI technologies in my practice of OMS	0 (0%)	12 (25.0%)	6 (12.5%)	15 (31.3%)	15 (31.3%)
Q5: AI makes completion of my work easier	8 (16.7%)	22 (45.8%)	13 (27.1%)	3 (6.3%)	2 (4.2%)
Q6: I feel adequately trained to use AI tools	0 (0%)	6 (12.5%)	4 (8.3%)	19 (39.6%)	19 (39.6%)
Q7: My clinic or institution is equipped to incorporate AI into clinical practice	0 (0%)	10 (20.8%)	9 (18.8%)	12 (25.0%)	16 (33.3%)

**Table 3:** Associations between demographic factors (gender, age and years of practice) and responses to Likert-scale survey questions.

Question	Female	Male	*p* value	Age ≤ 40	Age > 40	*p* value	1–5 years practice	6–15 years practice	> 15 years practice	*p* value
Section 2: Knowledge										
Q1			0.741			0.770				1.000
Disagree	7 (53.8%)	22 (62.9%)		17 (63.0%)	12 (57.1%)		9 (60.0%)	10 (58.8%)	10 (62.5%)	
Agree	6 (46.2%)	13 (37.1%)		10 (37.0%)	9 (42.9%)		6 (40.0%)	7 (41.2%)	6 (37.5%)	
Q2			0.335			1.000				0.393
Disagree	5 (38.5%)	20 (57.1%)		14 (51.9%)	11 (52.4%)		6 (40.0%)	11 (64.7%)	8 (50.0%)	
Agree	8 (61.5%)	15 (42.9%)		13 (48.1%)	10 (47.6%)		9 (60.0%)	6 (35.3%)	8 (50.0%)	
Q3			1.000			0.153				0.222
Disagree	11 (84.6%)	28 (80.0%)		24 (88.9%)	15 (71.4%)		14 (93.3%)	14 (82.4%)	11 (68.8%)	
Agree	2 (15.4%)	7 (20.0%)		3 (11.1%)	6 (28.6%)		1 (6.7%)	3 (17.6%)	5 (31.2%)	
Section 3: Attitudes										
Q1			1.000			0.004*				<0.001*
Disagree	0 (0.0%)	2 (5.7%)		0 (0.0%)	2 (9.5%)		0 (0.0%)	0 (0.0%)	2 (12.5%)	
Unsure	3 (23.1%)	7 (20.0%)		2 (7.4%)	8 (38.1%)		1 (6.7%)	1 (5.9%)	8 (50.0%)	
Agree	10 (76.9%)	26 (74.3%)		25 (92.6%)	11 (52.4%)		14 (93.3%)	16 (94.1%)	6 (37.5%)	
Q2			0.251			0.009*				0.024*
Disagree	0 (0.0%)	4 (11.4%)		0 (0.0%)	4 (19.0%)		0 (0.0%)	0 (0.0%)	4 (25.0%)	
Unsure	4 (30.8%)	5 (14.3%)		3 (11.1%)	6 (28.6%)		1 (6.7%)	4 (23.5%)	4 (25.0%)	
Agree	9 (69.2%)	26 (74.3%)		24 (88.9%)	11 (52.4%)		14 (93.3%)	13 (76.5%)	8 (50.0%)	
Q3			0.478			0.122				0.146
Disagree	1 (7.7%)	5 (14.3%)		2 (7.4%)	4 (19.0%)		1 (6.7%)	1 (5.9%)	4 (25.0%)	
Unsure	1 (7.7%)	8 (22.9%)		3 (11.1%)	6 (28.6%)		2 (13.3%)	2 (11.8%)	5 (31.2%)	
Agree	11 (84.6%)	22 (62.9%)		22 (81.5%)	11 (52.4%)		12 (80.0%)	14 (82.4%)	7 (43.8%)	
Q4			0.846			0.865				0.415
Disagree	11 (84.6%)	27 (77.1%)		22 (81.5%)	16 (76.2%)		11 (73.3%)	14 (82.4%)	13 (81.2%)	
Unsure	1 (7.7%)	6 (17.1%)		4 (14.8%)	3 (14.3%)		4 (26.7%)	1 (5.9%)	2 (12.5%)	
Agree	1 (7.7%)	2 (5.7%)		1 (3.7%)	2 (9.5%)		0 (0.0%)	2 (11.8%)	1 (6.2%)	
Q5			0.078			0.828				0.545
Disagree	2 (15.4%)	13 (37.1%)		9 (33.3%)	6 (28.6%)		7 (46.7%)	3 (17.6%)	5 (31.2%)	
Unsure	0 (0.0%)	5 (14.3%)		2 (7.4%)	3 (14.3%)		1 (6.7%)	2 (11.8%)	2 (12.5%)	
Agree	11 (84.6%)	17 (48.6%)		16 (59.3%)	12 (57.1%)		7 (46.7%)	12 (70.6%)	9 (56.2%)	
Section 4: Practices										
Q1			0.606			0.290				0.075
Disagree	4 (30.8%)	9 (25.7%)		5 (18.5%)	8 (38.1%)		1 (6.7%)	5 (29.4%)	7 (43.8%)	
Unsure	1 (7.7%)	1 (2.9%)		1 (3.7%)	1 (4.8%)		1 (6.7%)	0 (0.0%)	1 (6.2%)	
Agree	8 (61.5%)	25 (71.4%)		21 (77.8%)	12 (57.1%)		13 (86.7%)	12 (70.6%)	8 (50.0%)	
Q2			0.448			0.335				0.880
Disagree	7 (53.8%)	23 (65.7%)		15 (55.6%)	15 (71.4%)		9 (60.0%)	10 (58.8%)	11 (68.8%)	
Unsure	3 (23.1%)	3 (8.6%)		5 (18.5%)	1 (4.8%)		3 (20.0%)	2 (11.8%)	1 (6.2%)	
Agree	3 (23.1%)	9 (25.7%)		7 (25.9%)	5 (23.8%)		3 (20.0%)	5 (29.4%)	4 (25.0%)	
Q5			0.785			0.379				0.656
Disagree	2 (15.4%)	3 (8.6%)		2 (7.4%)	3 (14.3%)		1 (6.7%)	1 (5.9%)	3 (18.8%)	
Unsure	3 (23.1%)	10 (28.6%)		6 (22.2%)	7 (33.3%)		3 (20.0%)	5 (29.4%)	5 (31.2%)	
Agree	8 (61.5%)	22 (62.9%)		19 (70.4%)	11 (52.4%)		11 (73.3%)	11 (64.7%)	8 (50.0%)	
Q6			0.437			0.880				0.810
Disagree	9 (69.2%)	29 (82.9%)		21 (77.8%)	17 (81.0%)		11 (73.3%)	14 (82.4%)	13 (81.2%)	
Unsure	2 (15.4%)	2 (5.7%)		2 (7.4%)	2 (9.5%)		1 (6.7%)	1 (5.9%)	2 (12.5%)	
Agree	2 (15.4%)	4 (11.4%)		4 (14.8%)	2 (9.5%)		3 (20.0%)	2 (11.8%)	1 (6.2%)	
Q7			1.000			0.720				0.905
Disagree	8 (61.5%)	21 (60.0%)		15 (55.6%)	14 (66.7%)		8 (53.3%)	10 (58.8%)	11 (68.8%)	
Unsure	2 (15.4%)	7 (20.0%)		6 (22.2%)	3 (14.3%)		3 (20.0%)	4 (23.5%)	2 (12.5%)	
Agree	3 (23.1%)	7 (20.0%)		6 (22.2%)	4 (19.0%)		4 (26.7%)	3 (17.6%)	3 (18.8%)	

*Indicates statistically significant.

**Table 4:** Cronbach's alpha for sections 2–3 of the survey.

Section	Cronbach's alpha
2 (Knowledge)	0.76
3 (Attitudes)	0.71
4 (Practices)	0.78

When asked to list currently available AI technologies, 25 (52.1%) participants were able to list at least one related to OMS, of which 15 responses were related to examination and diagnosis, 7 were related to treatment planning, and 3 were for other uses. Outside of OMS, 24 (50.0%) participants could list a large language model, 5 (10.4%) mentioned robotics, and 5 (10.4%) mentioned speech-to-text and text-to-speech. 12 (25.0%) participants were unable to list any AI technology at all.

### Attitudes

Most participants were found to have positive attitudes about the use of AI in OMS (Table 2). The majority of respondents agreed that AI can enhance patient outcomes (75.0%), that AI should be integrated into clinical practice (72.9%), and that AI should be a part of OMS training (68.8%), while only a small minority of respondents (4.2-12.5%) answered “Strongly Disagree” or “Somewhat Disagree” to the same statements. On the other hand, 79.2% of participants disagreed that AI may replace surgeons in the future (79.2%), while 58.3% agreed that overuse of AI may cause loss of clinical skills. While there was no significant difference in responses by gender (*p* = 0.078–1.000), a significantly higher portion of respondents aged 40 and below agreed that AI can enhance patient outcomes (*p* = 0.004) and that AI should be integrated into clinical practice (*p* = 0.009) (Table 3). Similarly, significant differences in the proportion of “Agree” responses were found between participants with 5 or less, 6–15, and more than 15 years of clinical practice (*p* < 0.001 and *p* = 0.024 respectively). The Cronbach's alpha of 0.71 for the attitudes section indicated an acceptable level of consistency (Table 4).

Regarding the advantages and potential concerns of AI in OMS, 83.3% of respondents reported the advantage of increased efficiency, 72.9% reported reduced workload, 45.8% reported increased personalisation, and 6.3% reported AI did not have any advantages. While responses were similar across most demographic groups, a significantly higher proportion of participants aged 40 or less found increased personalisation to be an advantage (*p* = 0.013), and the proportion of participants who found AI to increase efficiency significantly differed by their number of years of clinical practice (*p* = 0.010) (Table 5). 77.1% of participants reported concerns of inaccurate diagnoses or treatment plans with the use of AI, 70.8% reported concerns of over-reliance, 41.7% reported privacy and security concerns, and 41.7% had concerns of increased healthcare costs. These findings were consistent across all demographic groups (Table 5).

**Table 5:** Associations between demographic factors (gender, age and years of practice) and responses to multi-response survey questions.

Question	Female	Male	*p*	Age ≤ 40	Age > 40	*p*	1–5 y	6–15 y	>15 y	*p*
Section 3: Attitudes										
Q6: Benefits										
Increased efficiency	11 (84.6%)	29 (82.9%)	1.000	9 (81.8%)	31 (83.8%)	1.000	13 (86.7%)	17 (100%)	10 (62.5%)	0.010*
Reduced workload	11 (84.6%)	24 (68.6%)	0.466	10 (90.9%)	25 (67.6%)	0.246	13 (86.7%)	11 (64.7%)	11 (68.8%)	0.355
Increased personalisation	8 (61.5%)	14 (40.0%)	0.210	9 (81.8%)	13 (35.1%)	0.013*	11 (73.3%)	6 (35.3%)	5 (31.2%)	0.050
No advantages	0 (0.0%)	3 (8.6%)	0.553	0 (0.0%)	3 (8.1%)	1.000	0 (0.0%)	0 (0.0%)	3 (18.8%)	0.059
Q7: Concerns										
Privacy/security	5 (38.5%)	15 (42.9%)	1.000	4 (36.4%)	16 (43.2%)	0.741	5 (33.3%)	9 (52.9%)	6 (37.5%)	0.555
Inaccuracy	9 (69.2%)	28 (80.0%)	0.458	7 (63.6%)	30 (81.1%)	0.246	12 (80.0%)	12 (70.6%)	13 (81.2%)	0.755
Over-reliance	4 (30.8%)	16 (45.7%)	0.512	7 (63.6%)	26 (70.3%)	0.720	9 (60.0%)	11 (64.7%)	13 (81.2%)	0.445
Increased costs	11 (84.6%)	22 (62.9%)	0.182	4 (36.4%)	16 (43.2%)	0.741	8 (53.3%)	4 (23.5%)	8 (50.0%)	0.175
Section 4: Practices										
Q3: Uses in OMS										
Diagnosis	5 (38.5%)	17 (48.6%)	0.746	6 (54.5%)	16 (43.2%)	0.732	10 (66.7%)	9 (52.9%)	3 (18.8%)	0.020*
Education	6 (46.2%)	14 (40.0%)	0.750	6 (54.5%)	14 (37.8%)	0.488	8 (53.3%)	9 (52.9%)	3 (18.8%)	0.091
Self-directed learning	4 (30.8%)	14 (40.0%)	0.740	6 (54.5%)	12 (32.4%)	0.288	8 (53.3%)	5 (29.4%)	5 (31.2%)	0.341
Treatment planning	3 (23.1%)	15 (42.9%)	0.317	4 (36.4%)	14 (37.8%)	1.000	5 (33.3%)	7 (41.2%)	6 (37.5%)	0.934
Intraoperative use	1 (7.7%)	6 (17.1%)	0.656	2 (18.2%)	5 (13.5%)	0.653	4 (26.7%)	1 (5.9%)	2 (12.5%)	0.227
Have/will not use	3 (23.1%)	7 (20.0%)	1.000	0 (0.0%)	10 (27.0%)	0.089	0 (0.0%)	3 (17.6%)	7 (43.8%)	0.009*
Q4: Use in Subspecialties										
Dentofacial deformities	5 (41.7%)	18 (51.4%)	0.740	5 (50.0%)	18 (48.6%)	1.000	8 (57.1%)	9 (52.9%)	6 (37.5%)	0.594
Dentoalveolar surgery	6 (50.0%)	7 (20.0%)	0.065	2 (20.0%)	9 (24.3%)	1.000	5 (35.7%)	4 (23.5%)	2 (12.5%)	0.358
Surgical pathology	2 (16.7%)	9 (25.7%)	0.703	3 (30.0%)	13 (35.1%)	1.000	4 (28.6%)	7 (41.2%)	5 (31.2%)	0.800
Maxillofacial trauma	3 (25.0%)	13 (37.1%)	0.505	2 (20.0%)	10 (27.0%)	1.000	4 (28.6%)	4 (23.5%)	4 (25.0%)	1.000
Implant surgery	2 (16.7%)	10 (28.6%)	0.703	1 (10.0%)	17 (45.9%)	0.065	3 (21.4%)	8 (47.1%)	7 (43.8%)	0.357
TMJ surgery	3 (25.0%)	15 (42.9%)	0.324	0 (0.0%)	5 (13.5%)	0.569	1 (7.1%)	1 (5.9%)	3 (18.8%)	0.519
Have/will not use	1 (8.3%)	4 (11.4%)	1.000	3 (30.0%)	10 (27.0%)	1.000	3 (21.4%)	3 (17.6%)	7 (43.8%)	0.225

*Indicates statistically significant.

### Practices

Although most participants reported having used AI outside of work (68.8%), only 25.0% reported having used it within the field of OMS. While 62.5% expressed that AI can make the completion of their work easier, most reported that they felt inadequately trained to do so (79.2%), and that their clinic was not equipped to do so (58.3%) (Table 2). There was no significant difference in responses by gender (*p* = 0.437-1.000), age (*p* = 0.290–0.880) or number of years of practice (*p* = 0.075–0.905) (Table 3). The Cronbach's alpha of 0.78 for the practices section indicated an acceptable level of consistency (Table 4).

Of the potential uses of AI in OMS, respondents stated that they had used or considered using AI most frequently for diagnosis (45.8%), followed by patient or student education (41.7%), self-directed learning (37.5%), treatment planning (37.5%), and as an intraoperative aid (16.7%). Responses were only significantly different between participants with different years of clinical practice (*p* = 0.020) in their consideration of use of AI for diagnosis (Table 5). 11 (22.9%) respondents stated they had never used nor considered using AI in practice; this was found in significantly higher proportion in clinicians with more than 15 years in practice (*p* = 0.009). The subspecialty of dentofacial deformities was considered for the integration of AI most frequently (50.0%), followed by implant surgery (39.6%) and surgical pathology (35.4%). No differences were found between preference of use in any subspecialty and demographic factors (*p* > 0.05).

### Thematic analysis

For the open-ended question on what resources were needed for better integration of AI into OMS, four themes were identified. These themes are: (1) Access to training, (2) structuring, (3) funding, and (4) technological refinement. Ten (20.8%) responses fit into the first theme of access to training, with respondents stating that clinicians would benefit from structured training programs, courses, and access to relevant software. Five (10.4%) of respondents mentioned the second theme of the need for better structuring and workflows to facilitate smooth implementation. Under this theme, respondents mentioned the potential benefits of improved institutional and department workflows and sorting out of regulatory issues regarding consent and privacy prior to widespread implementation. For the third theme of funding, 5 (10.4%) of responses emphasised the need for better monetary support in the form of government or institutional grants to reduce the barriers to entry for integration. Lastly, 2 (4.2%) respondents mentioned the final theme of technological refinement. One respondent suggested the creation of a country-specific database to further refine AI models for better applicability to the local population, while the other mentioned their hesitance to use AI tools until the tools show better results.

## Discussion

There is widespread acknowledgement that AI can play a significant role in the diagnosis, prognostication, treatment planning and even intraoperative management of surgical patients (11). To keep up with the advances in medical technology, it is thus important to understand the current climate and potential challenges that OMS clinicians are facing to reduce the barriers to entry to integrating AI.

The responses from the knowledge section of the survey found that only half of the participants knew of the uses of AI in OMS. Similarly, only half were able to name an AI technology that can be used in practice, while a quarter were unable to name any AI technology at all. These levels of knowledge are similar to those found in similar studies done with other healthcare workers and healthcare students (7, 12, 13). In this study, with no differences found between the various demographic groups, the overall lacklustre level of knowledge could be attributed to a lack of formal training opportunities for clinicians to learn more about AI and its potential uses; more than 80% of respondents had never attended any form of AI-related training. This finding, however, does not seem to be confined to OMS; a survey of radiologists found that almost 70% did not receive any AI training (14). While there has been a significant increase in the number of published articles on the development of AI models in surgery (15), the exposure of these new developments does not seem to be reaching a good number of clinicians in our population.

Most responses from the attitudes section of the survey were optimistic about the potential role of AI in OMS. A majority of participants “strongly agreed” or “agreed” that AI could enhance patient outcomes and should be integrated into both practice and training. Similar sentiments were found in previously published studies of nursing staff and other healthcare workers, where most participants felt AI could aid diagnosis and treatment planning and was essential in medicine and nursing (12, 16). However, unlike these studies, which reported concern of AI replacing their jobs in about half of the participants, only 6.3% of respondents in this study were concerned about AI replacing them in the future. While there is confidence that AI will not be taking over the job scope of OMS any time soon, more than 50% of participants still cautioned against overuse that may result in the loss of clinical skills.

Participants who were younger than the mean age of 40.8 and those with fewer years of clinical experience tended towards more positive attitudes towards AI in OMS. Although only the statements “AI can enhance patient outcomes” and “AI should be integrated into practice” reached statistical significance, many statistically insignificant results still revealed higher proportions of positive attitudes towards AI in younger participants with fewer years in practice. The statement “AI should be a part of OMS training”, for example, resonated with 81.5% of participants aged 40 and below and 80% of participants with 1–5 years of clinical practice, but only 52.4% of participants above 40 and 43.8% of participants with more than 15 years of practice. A significantly higher proportion of participants with more than 15 years of practice also stated that they have and will not use AI in their practice. Given that the younger respondents naturally have fewer years in practice, this trend is consistent with the commonly found notion that younger generations are more likely to embrace technologies like AI (17).

In the practice section, the issues of a lack of training and experience were highlighted by many participants. Although almost two-thirds of participants felt that AI could make work easier, the majority had not tried to use AI in their work at all, and expressed that they were not adequately equipped with the necessary skills to incorporate AI into daily practice; this was the consensus across all demographic groups. Along the same vein, a combined 30% of respondents called for improved access to training and more concrete workflows to smooth the uptake of AI in practice. Similar issues and mindsets are seen elsewhere within the medical field, with medical schools lacking the expertise required to incorporate AI into their curriculum, and recommendations by healthcare workers to collaborate with software developers and start initiatives to increase awareness of the uses of AI (14, 18). Moving forward, integration of AI with OMS can perhaps start with introducing it into the postgraduate (or even undergraduate) training curriculum, to familiarise clinicians with AI from the start and minimise the barrier to entry that stems from unfamiliarity.

### Outstanding concerns

Other valid concerns of the participants in this study can be divided into three broad groups: concerns regarding inaccurate diagnoses and treatment plans, privacy and security concerns, and increased healthcare costs. The four suggestions proposed by the participants to improve access to training, create better workflows for implementation, increase funding and further refine and optimise AI models are spot on in addressing the perceived issues revolving around using AI in OMS.

The possibility of inaccurate diagnoses and treatment plans was the most common concern among participants, with a small group even imploring more development and refinement of AI models before incorporating them into practice. The ability of AI to correctly answer OMS-related questions is still not ideal, with a study of large language models finding a mean score of just 62.5% or a B grade on OMS examination questions (19). However, emerging evidence shows the high diagnostic accuracy of AI models in detecting pathology using clinical records, photographs, radiographs or histopathological slides, and in the prognostication of oral diseases (20–23) Nevertheless, the repercussions of an incorrect diagnosis have led some to call for rigorous validation processes before implementation (24). Overall, overcoming this concern will require two improvements: the enhancement of AI models to optimise accuracy, and the spread of understanding that AI is not meant to replace the clinician, but to instead aid them in their tasks.

Concerns regarding security and a breach of patient privacy were expressed by almost half of the respondents. This concern is a major one, since large amounts of data are fed to and processed by AI models for their training and validation. Breaches in patient privacy during both model development and utilisation can occur, since acts of governance such as the Health Insurance Portability and Accountability Act (HIPAA) currently do not have clearly established guidelines covering AI-related technologies (25). Fears that large tech companies like Google can re-identify de-identified data through triangulation with other data sets are also not baseless, as re-identification has been proven successful before (26, 27), and lawsuits because of this have been filed (28). Resolving this issue necessitates two solutions. First, consideration can be made to develop future AI models with realistic synthetic patient data created by generative models instead of real patient data (29), to obviate the need for real patient data where possible. Second, regulations regarding patient data need to be revisited and refined to improve privacy legislation specific to the use of AI in healthcare.

On a similar note, although other ethical considerations of the integration of AI into OMS were not mentioned by any of our participants, it is a potential issue that needs discussion. As much as AI has the potential to improve patient outcomes, training and research, its implementation should still uphold transparency, informed consent of its use, and patient autonomy to disclose their health information and make decisions relating to their care (30). Professionalism must be maintained through a declaration of its use in practice and research to both patients and colleagues. Furthermore, it is paramount to understand that AI in its current stage should serve to complement, and not replace, the human touch still required in patient care, education and research. A review by Rokshad and colleagues highlighted a framework that can be implemented in the future refinement of AI models in dental practice and research; this framework addresses ethical challenges based on the eleven ethical pillars of transparency, diversity, wellness, respect of autonomy, privacy, accountability, equity, prudence, sustainability, solidarity and governance, and are a good guide to understanding how to best protect our patients' interests in the integration of AI (31).

Lastly, almost half of the respondents in this study cited the concern of potentially increased healthcare costs. Interestingly, this was in contrast to a similar study of nurses, where most participants thought that AI could instead reduce healthcare costs (16). While fears that the cost of development of AI technologies may be carried forward to the patient's bill, a review of 200 studies in 2022 has instead shown that integrating AI into healthcare reaps significant cost savings (32). This can be attributed to reduced diagnostic and treatment time and improved efficiency that increases with the number of years of its use. While no cost-effectiveness studies specific to OMS have been published, the abovementioned cost savings have been reported in other aspects of dentistry, such as its use for dental caries detection or the early identification of oral mucosal lesions (33, 34). To prevent increased individual patient costs, it may be in the interest of governments and institutions to provide funding for the development of these models. Other ways to reduce healthcare costs include pruning of the models to eliminate unnecessary components, as well as developing explainable AI models which incorporate a feedback loop to improve their usability and long-term sustainability (32).

This cross-sectional study is the first one conducted to evaluate the knowledge, attitudes and practices of specialists and trainees in the field of OMS, and while similar views are shared with other healthcare workers, gaining an understanding of the attitudes and concerns of people in our fraternity allows us to form concrete solutions to outstanding issues. However, the study has its limitations. First, due to the small population size of OMS in Singapore, the study may be underpowered. While 55 responses were initially recorded, the ultimate sample size was below our target sample size of 52, as 7 responses were incomplete and had to be excluded. As there is only one postgraduate training program in the country, the mindsets of trainees could also be skewed, thus potentially limiting the applicability of their perspectives to other populations. Consequently, the small sample size may imply results that are less generalisable and robust. Addressing this limitation will require multi-centre execution across various countries in the region to increase future sample sizes.

Convenience sampling was used for this study due to the limited manpower available in this project to perform more sophisticated sample methods (e.g., systematic or stratified sampling). While convenience sampling is simple to perform, it may introduce bias towards people who use technologies like social media more often and skew the results to favour technologies like AI. Fortunately, the small OMS population size and high digital literacy in Singapore meant that dissemination via emails and social media channels of local professional organisations was likely to reach the vast majority of OMS clinicians in the country.

Furthermore, the views reported in this study are specific to our population of mostly ethnically Southern Chinese clinicians, and may reflect different levels of acceptance towards AI compared to Caucasian, African or other ethnic groups (35). Finally, it is important to acknowledge that the results of this study only reflect the current population of clinicians in OMS. The views of clinicians may be very different ten years from now, due to the likely further advancement of AI systems and possibly legislative reforms and institutional shifts towards AI-driven care that can make its acceptance more widespread.

## Conclusion

While the attitudes of OMS specialists and trainees in Singapore on AI are generally positive, the levels of knowledge and practice leave room for improvement. The feedback on potential areas for improvement necessitates further technological and policy refinement prior to the inevitable integration of AI into daily practice and education.

## Data Availability

The raw data supporting the conclusions of this article will be made available by the authors, without undue reservation.
